# Validation and Calibration of a Computer Simulation Model of Pediatric HIV Infection

**DOI:** 10.1371/journal.pone.0083389

**Published:** 2013-12-13

**Authors:** Andrea L. Ciaranello, Bethany L. Morris, Rochelle P. Walensky, Milton C. Weinstein, Samuel Ayaya, Kathleen Doherty, Valeriane Leroy, Taige Hou, Sophie Desmonde, Zhigang Lu, Farzad Noubary, Kunjal Patel, Lynn Ramirez-Avila, Elena Losina, George R. Seage III, Kenneth A. Freedberg

**Affiliations:** 1 Division of Infectious Diseases, Massachusetts General Hospital, Boston, Massachusetts, United States of America; 2 Division of General Medicine, Massachusetts General Hospital, Boston, Massachusetts, United States of America; 3 Medical Practice Evaluation Center, Massachusetts General Hospital, Boston, Massachusetts, United States of America; 4 Division of Infectious Diseases, Brigham and Women's Hospital, Boston, Massachusetts, United States of America; 5 Department of Medicine, and Department of Orthopedic Surgery, Brigham and Women's Hospital, Boston, Massachusetts, United States of America; 6 The Center for AIDS Research, Harvard University, Boston, Massachusetts, United States of America; 7 The Department of Health Policy and Management, Harvard School of Public Health, Boston, Massachusetts, United States of America; 8 The Department of Epidemiology, Harvard School of Public Health, Boston, Massachusetts, United States of America; 9 Department of Child Health and Pediatrics, Moi University, Eldoret, Kenya; 10 Inserm, Unit 897, Institut de Santé Publique et de Développement, Université Bordeaux Segalen 2, Bordeaux, France; 11 Children's Hospital Boston, Boston, Massachusetts, United States of America; 12 Department of Pediatrics, Division of Pediatric Infectious Diseases, University of California Los Angeles, Los Angeles, California, United States of America; 13 The Department of Biostatistics, Boston University School of Public Health, Boston, Massachusetts, United States of America; 14 The Department of Epidemiology, Boston University School of Public Health, Boston, Massachusetts, United States of America; University of North Carolina School of Medicine, United States of America

## Abstract

**Background:**

Computer simulation models can project long-term patient outcomes and inform health policy. We internally validated and then calibrated a model of HIV disease in children before initiation of antiretroviral therapy to provide a framework against which to compare the impact of pediatric HIV treatment strategies.

**Methods:**

We developed a patient-level (Monte Carlo) model of HIV progression among untreated children <5 years of age, using the Cost-Effectiveness of Preventing AIDS Complications model framework: the CEPAC-Pediatric model. We populated the model with data on opportunistic infection and mortality risks from the International Epidemiologic Database to Evaluate AIDS (IeDEA), with mean CD4% at birth (42%) and mean CD4% decline (1.4%/month) from the Women and Infants’ Transmission Study (WITS). We internally validated the model by varying WITS-derived CD4% data, comparing the corresponding model-generated survival curves to empirical survival curves from IeDEA, and identifying best-fitting parameter sets as those with a root-mean square error (RMSE) <0.01. We then calibrated the model to other African settings by systematically varying immunologic and HIV mortality-related input parameters. Model-generated survival curves for children aged 0-60 months were compared, again using RMSE, to UNAIDS data from >1,300 untreated, HIV-infected African children.

**Results:**

In internal validation analyses, model-generated survival curves fit IeDEA data well; modeled and observed survival at 16 months of age were 91.2% and 91.1%, respectively. RMSE varied widely with variations in CD4% parameters; the best fitting parameter set (RMSE = 0.00423) resulted when CD4% was 45% at birth and declined by 6%/month (ages 0-3 months) and 0.3%/month (ages >3 months). In calibration analyses, increases in IeDEA-derived mortality risks were necessary to fit UNAIDS survival data.

**Conclusions:**

The CEPAC-Pediatric model performed well in internal validation analyses. Increases in modeled mortality risks required to match UNAIDS data highlight the importance of pre-enrollment mortality in many pediatric cohort studies.

## Introduction

Key clinical and operational research questions related to prevention, diagnosis, and therapy for HIV-infected children remain unanswered. For example, estimates of the long-term outcomes of immediate versus deferred ART initiation strategies for children 0-5 years of age, the cost-effectiveness of alternative first-line ART regimens, and the relative value of early infant diagnosis algorithms are needed to inform HIV care guidelines [1–3]. 

While clinical trials and cohort studies will continue to address these questions, computer simulation models comprise important adjuncts to these more traditional research methods. Models can integrate available data, project long-term clinical and economic outcomes beyond study periods, identify influential parameters for which additional data are needed, and inform care and treatment guidelines [4–14]. To date, three published analyses have reported on simulation models of HIV-infected children: a Markov model used to evaluate the cost-effectiveness of cotrimoxazole prophylaxis and of laboratory monitoring of ART, and a decision-analytic model of strategies for early infant diagnosis [15–17].

The Cost-Effectiveness of Preventing AIDS Complications (CEPAC) model is a validated, individual patient-level (Monte Carlo) simulation of HIV disease in adults that has informed HIV testing and treatment policy in the United States and internationally [4,5,18–22]. Building on the adult CEPAC model platform, we developed a simulation model of HIV disease in infants and children <5 years of age, the CEPAC-Pediatric model, to address policy questions related to prevention, diagnosis and treatment of pediatric HIV. The objectives of this analysis were to internally validate the structure of the CEPAC-Pediatric model; to calibrate the model to survival data from untreated HIV-infected children in sub-Saharan Africa; and to describe this work in an open-access forum using recommended reporting practices [23–25].

## Methods

### Ethics

This work was approved by the Partners Healthcare IRB.

### Analytic overview

We developed a microsimulation model of pediatric HIV disease progression, the CEPAC-Pediatric model. As in the adult CEPAC model, clinical events are first simulated and validated in the absence of ART (a "natural history" model), in order to describe disease progression in the absence of ART and to provide a framework against which to compare the impact of HIV treatment [4,5]. In collaboration with the International Epidemiologic Databases to Evaluate AIDS (IeDEA) consortium [26,27], we derived model input parameters for the CEPAC-Pediatric model, reflecting outcomes in HIV-infected children prior to the initiation of ART. These model input data included rates of WHO Stage 3 and Stage 4 clinical events, tuberculosis (TB), and mortality [28], stratified by age and CD4%.

Internal model validation is a formal methodology to assess the validity of model structure. In internal calibration, the empiric data values used in the modeling analysis ("model inputs") are compared to model-generated results ("model outputs"), in order to assess model performance for analyses related to a single data set [19,24,25,29–31]. We conducted internal model validation by comparing model-generated results to the clinical event and mortality risks observed in the same IeDEA cohort that contributed model input data. For internal validation, selected immunologic parameters that were not available from IeDEA were based on data from the Women and Infants' Transmission Study (WITS) [32–34]. 

Model calibration is a methodology distinct from validation. In model calibration, sometimes referred to as “model fitting,” investigators identify the values for key data parameters that will allow model projections to match empiric observations. Calibration seeks to explicitly modify model input parameters, in order to make the model useful for predicting outcomes in cohorts or datasets distinct from the dataset used in internal validation [19,23–25,29]. The IeDEA East African cohort represents a highly selected population of children with excellent access to HIV care. In order to produce analyses more generalizable to other African settings, we identified data parameter sets that allowed model output to match published survival curves from a pooled UNAIDS analysis of >1,300 untreated, perinatally HIV-infected children in eight sub-Saharan African countries [30,35–38].

### Model structure

**Figure 1:**
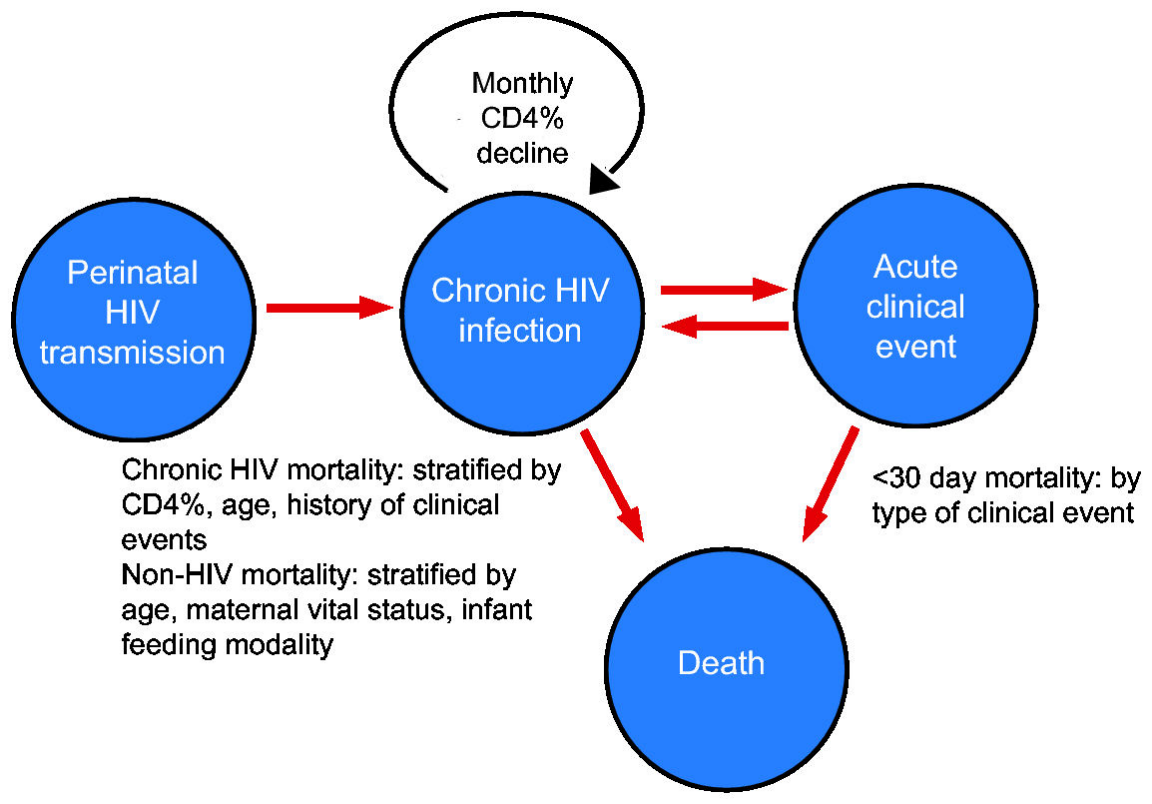
CEPAC-Pediatric model structure. A schematic of the Cost-Effectiveness of Preventing AIDS Complications (CEPAC)-Pediatric natural history model (see Methods for details).

The CEPAC-Pediatric model is a first-order, patient-level Monte Carlo simulation model (Figure 1). Infants enter the natural history model at birth, and are assumed to have been HIV-infected either *in utero* or during delivery (intrapartum). A random number generator is used to draw from user-specified distributions of maternal HIV status (CD4 ≤350/μL or >350/μL; receiving or not receiving ART), PMTCT exposure; breastfeeding or replacement feeding; and infant CD4% at birth (percentage of total lymphocytes that are CD4+ cells). We modeled CD4% as the primary immunologic measure for children <5 years of age because absolute CD4 count declines dramatically with age, even in the absence of HIV infection, and CD4% is therefore a more stable marker of immune function as children age [3]. In the absence of ART, each simulated child's CD4% declines monthly at a user-specified rate until they reach age five. Older children, adolescents, and adults can be simulated in dedicated analyses using the CEPAC adult model; in conjunction with the CEPAC-Pediatric model, this permits projections over the lifetimes of HIV-infected children [4–6]. 

Disease progression in the CEPAC-Pediatric model is characterized by monthly transitions among health states, including chronic HIV infection, acute clinical events, and death (Figure 1). Transitions between these health states depend on current age (0-2, 3-5, 6-8, 9-11, 12-17, 18-23, 24-35, 36-47, and 48-59 months) and CD4% (<15%, 15-24%, and ≥25%) during each month of the simulation. Simulated patients face monthly risks of up to 10 types of acute "clinical events," including opportunistic infections and other HIV-related illnesses. For this analysis, reflecting available IeDEA data, we modeled 3 mutually exclusive categories of clinical events: WHO Stage 3 events (WHO 3, excluding pulmonary and lymph node tuberculosis (TB)), WHO Stage 4 events (WHO 4, excluding extrapulmonary TB), and TB events (at any anatomic site) [28]. 

The CEPAC-Pediatric model simulates three types of mortality. First, children with no history of acute clinical event face a monthly risk of HIV-related death ("chronic HIV mortality"), stratified by current age and CD4%. Second, children who experience a clinical event face "acute mortality" risks in the first 30 days post-event, stratified by current age. After this 30-day "acute mortality" period, children return to “chronic HIV mortality,” though with increased monthly risks compared to age/CD4%-matched children without a history of clinical events. Third, in addition to HIV-related mortality, the model includes a monthly risk of "non-AIDS death," derived from UNAIDS age- and sex-adjusted, country-specific mortality rates that exclude the impact of HIV [39].

For each simulated infant, the model tracks clinical events, changes in CD4%, and the amount of time spent in each health state. After an individual simulated patient has died, the next infant enters the model. Large cohorts (often 1 million-10 million patients) are simulated in order to generate stable model outcomes. Once the entire cohort has been simulated, summary statistics are tallied, including number and type of clinical events and the proportion alive each month. Additional information about CEPAC-Pediatric model structure, data sources, and procedures for initiating new collaborative projects are available at web2.research.partners.org/cepac/model.html.

### IeDEA East Africa natural history model input data (Tables 1 and 2)

**Table 1:** Selected model input parameters in the CEPAC-Pediatric natural history model for internal validation analyses.

**Data from the IeDEA East Africa cohort** [28]		**Value**			
**Monthly risk of clinical events (%) ^a^**		Infants <6m of age		Children ≥6m of age	
WHO Stage 3 event		5.2-7.8		3.3-11.6	
WHO Stage 4 event		1.6-3.5		1.4-6.4	
Tuberculosis event		0.5-1.1		0.8-3.8	
**Risk of death within 30 days of clinical event (%)**					
After WHO Stage 3 or 4 event		3.4			
After TB event		2.8			
**Monthly risk of death in infants and children with no history of clinical event (%)**					
CD4% < 15		0.4			
CD4% 15-24		0.4			
CD4% ≥ 25		0.3			
**Monthly risk of death in infants and children with history of clinical event (%, occurring >30 days post-event))**					
CD4% < 15		2.4			
CD4% 15-24		0.8			
CD4% ≥ 25		0.4			
**Data from WITS [33]^b^**	**Value**		**Range evaluated in internal validation analyses**		
Initial CD4% distribution at birth (mean, SD)	42.0% (9.4%)		42.0% - 50.0% **^c^**		
Monthly rate of CD4% decline	1.4%		0.3% - 8.0% **^c^**		

**IeDEA**: International Epidemiologic Databases for the Evaluation of AIDS; **WHO**: World Health Organization; **TB**: tuberculosis; **WITS**: Women and Infants Transmission Study.

a. WHO Stage 4, Stage 4, and TB events defined according to WHO classifications for HIV disease staging in children [3].

b. The publicly available WITS dataset includes 193 perinatally HIV-infected children (positive HIV co-culture or PCR by 4-6 weeks of age), with a median of 5.2 months of follow-up prior to initiation of 3-drug ART (Interquartile Range (IQR): 2.1-12.1 months; AZT monotherapy was permitted during the follow-up period) [33]. Of the 193 perinatally HIV-infected children included in the WITS dataset, 180 (93%) had at least one CD4% measurement before ART initiation, 152 (79%) had at least two values, and 121 (63%) had at least three; the first recorded CD4% was observed at a median age of 5.0 days (IQR: 1.0-29.0 days)

c. See derivation of ranges for sensitivity analyses in Methods.

**Table 2:** Selected model input parameters in the CEPAC-Pediatric natural history model for calibration analyses.

**Data from the IeDEA East Africa cohort** [28]	**Value**	**Range evaluated in calibration analyses**
**Monthly risk of clinical events (%) ^a^**		
Identical to data parameters used in internal validation analyses, above		Not varied for calibration analyses
**Risk of death within 30 days of clinical event (%)**		Range, 0.5-5 X IeDEA risk
After WHO Stage 3 or 4 event	3.4	1.7-16.8
After TB event	2.8	1.4-13.9
**Monthly risk of death in infants and children with no history of clinical event (%)**		Range, 0.2-20 X IeDEA risk
CD4% < 15	0.4	0.08-8.3
CD4% 15-24	0.4	0.07-7.2
CD4% ≥ 25	0.3	0.06-6.2
**Monthly risk of death in infants and children with history of clinical event (%, occurring >30 days post-event)**		Range, 0.2-20 X IeDEA risk
CD4% < 15	2.4	0.5-48.0
CD4% 15-24	0.8	0.2-16.7
CD4% ≥ 25	0.4	0.08-7.9
**Data from WITS [33]^b^**	**Value**	**Range evaluated in calibration analyses**
Initial CD4% distribution at birth (mean, SD)	42.0% (9.4%)	42.0% - 50.0% **^c^**
Monthly rate of CD4% decline	1.4%	0.3% - 8.0% **^c^**
**Data from UNAIDS** [39]	**Value**	**Range evaluated in calibration analyses**
**HIV-deleted mortality for Burkina Faso, Côte d'Ivoire, Kenya, South Africa, Tanzania, and Uganda ^d^ (monthly risks**)		
0-11 months	0.41-0.49%	Not varied for calibration analyses
12-59 months	0.04-0.05%	Not varied for calibration analyses

**IeDEA**: International Epidemiologic Databases for the Evaluation of AIDS; **WHO**: World Health Organization; **TB**: tuberculosis; **WITS**: Women and Infants Transmission Study.

a. WHO Stage 4, Stage 4, and TB events defined according to WHO classifications for HIV disease staging in children [3].

b. The publicly available WITS dataset includes 193 perinatally HIV-infected children (positive HIV co-culture or PCR by 4-6 weeks of age), with a median of 5.2 months of follow-up prior to initiation of 3-drug ART (Interquartile Range (IQR): 2.1-12.1 months; AZT monotherapy was permitted during the follow-up period) [33]. Of the 193 perinatally HIV-infected children included in the WITS dataset, 180 (93%) had at least one CD4% measurement before ART initiation, 152 (79%) had at least two values, and 121 (63%) had at least three; the first recorded CD4% was observed at a median age of 5.0 days (IQR: 1.0-29.0 days)

c. See derivation of ranges for sensitivity analyses in Methods.

d. UNAIDS HIV-deleted mortality rates from these eight countries were weighted by the proportion of children from each country included in the UNAIDS pooled analysis used as a calibration target [35,36].

IeDEA is an international consortium of AIDS care and treatment centers [26,27,40]. In previous work, we estimated incidence rates of first clinical event (WHO3, WHO4 and TB), acute mortality (<30 days after clinical event), and chronic HIV mortality among untreated, HIV-infected children at seven clinical sites in the IeDEA East Africa region [28]. Additional details about the IeDEA East Africa sites, as well as methods for derivation of model input parameters, have previously been described [28,41]. 

#### Baseline cohort characteristics and clinical event risks

In the IeDEA East African cohort, all children enrolled in care prior to 12 months of age (median: 5 months); 52% were female [28]. We translated observed IeDEA event rates into monthly transition probabilities (risks), stratified by age and CD4% (Table 1). In children <6 months old, clinical event risks ranged from 5.2-7.8%/month for WHO3, 1.6-3.5%/month for WHO4, and 0.5-1.1%/month for TB. For children ≥6 months of age, clinical event risks ranged from 3.3-11.6%/month for WHO3, 1.4-6.4%/month for WHO4, and 0.8-3.8%/month for TB (Table 1). Modeled risks of subsequent clinical events were assumed to be equal to risks of first events, within each age and CD4% stratum. 

#### Mortality risks

For children with no history of clinical events, monthly risks of chronic HIV mortality ranged by CD4% from 0.3-0.4%. For children with a clinical event, the 30-day risk of acute mortality following a WHO3 or WHO4 event was 3.4%, and the risk following TB events was 2.8%. After the 30-day period post-event, monthly risks of "chronic HIV mortality" ranged by CD4% from 0.4-2.4%. Non-AIDS death risks (reflecting age- and sex-adjusted mortality rates) were held at zero for internal validation analyses, since all observed deaths in the IeDEA cohort were coded as HIV-related and thus considered either acute or chronic HIV-related mortality. For calibration analyses, "non-AIDS" mortality rates were from UNAIDS HIV-deleted life tables for the eight sub-Saharan countries which were included in the study (Table 2) [35,36,39]. 

### WITS natural history model input data (Tables 1 and 2)

#### Immunologic data

Because IeDEA lacked adequate longitudinal CD4 data, we derived CD4% at birth and rate of monthly CD4% decline from the US-based WITS, a longitudinal cohort study (1990-2006) of HIV-infected women and their infants during pregnancy and the post-partum period [32–34]. Using a mixed effects model for the primary analysis, we estimated a mean CD4% at birth of 42.0% (standard deviation, 9.4%), and a monthly CD4% decline of 1.4%/month prior to ART initiation [42]. In a secondary analysis in which CD4% was permitted to decline by different rates in months 0-2 and 3+ of life, we estimated a mean CD4% of 50.0% at birth, monthly decline of 6.4%/month for months 0-2, and 0.3%/month in months 3+. Due to high variability around the point estimate for this latter variable, likely due to small numbers of CD4% data in older infants, we used these results to inform the ranges of CD4% parameters for sensitivity analyses, rather than for the primary analysis.

### Internal model validation: Comparison of model-generated results to empiric data from the IeDEA East African region

#### Population and follow-up time

For internal validation analyses, we simulated a population of HIV-infected infants from birth (assuming intrauterine or intrapartum infection), with clinical characteristics of patients in the IeDEA cohort. To most closely match the observed IeDEA data, we evaluated model-generated results for children from 5-16 months of age, reflecting a median age at enrollment in the IeDEA cohort of 5 months and a median of 11 months follow-up [28]. 

#### Internal validation: survival outcomes

We compared model-generated survival curves from 5 to 16 months after birth to Kaplan-Meier survival curves directly from the IeDEA East African regional data. We first assessed model results using base-case parameter estimates. We then performed two-way sensitivity analyses in which we simultaneously varied the two parameters from WITS (CD4% at birth and monthly CD4% decline). First, CD4% at birth was varied in 1.0% increments from 42% (the result in the primary WITS analysis) to 50% (the result from the secondary WITS analysis). This range includes the value of 47%, which was the mean percentage recorded in the first 1-2 days of life in a study in Durban, South Africa [43,44]. Second, the monthly rate of CD4% decline was varied from 0.3% (the lowest value from the secondary WITS sensitivity analysis) to 8.0% (an average of published values in the first three months of life [43–45]). To reflect observations that CD4% may decline more rapidly in the first few months of life [43,44], we permitted CD4% to decline at different rates for "younger" and "older" infants. We defined "younger" and "older" age groups using threshold values of 3, 6, or 12 months of age, and examined all combinations of CD4% at birth and monthly CD4% decline in which CD4% decline was faster in “younger” compared to “older” children.

For each parameter set, we compared model-based survival curves to the empiric IeDEA survival curves at each month of the simulation using root-mean-square error (RMSE) [30]. RMSE was calculated as the square root of the average of the squared difference between observed and projected survival proportions at each month over the course of the simulation (5-16 months). We defined the best-fitting survival curves as those with a RMSE <0.01. This method was chosen because it is intuitive, computationally feasible with complex models, and appropriate for data drawn primarily from a single source [23–25,30].

#### Internal validation: clinical event risks

In addition to examining survival results, we also compared the model-generated rates of clinical events to the observed rates in the IeDEA cohort. Because model-based analyses do not rely on a single convention for comparing model results to data [24,25], we defined a good-fitting result as one where model-projected incidence rates were within 10-15% (relative) of observed data, based on previous work [5]. To reflect as closely as possible the IeDEA clinical cohort, simulated infants entered the model at birth, with the initial CD4% distribution and rates of monthly CD4% decline identified in the best-fitting parameter set in the internal validation survival analyses described above. Model-based incidence rates for first clinical events between 5 and 16 months of age were projected for infants. Number of events and time at risk are not stratified by CD4% in the current model output, because they were not anticipated for use in future policy analyses. To directly compare model output with IeDEA data, we re-analyzed IeDEA event rates for all children (combining all CD4% strata) at ages <6 and ≥6 months of age.

### Model calibration: Comparison of model-generated results to published pre-ART survival curves

#### Calibration targets, modeled population, and follow-up time

Following internal validation of the CEPAC-Pediatric model, we compared model-generated results to survival data reported in a pooled UNAIDS analysis of perinatally HIV-infected children in sub-Saharan Africa [35–38]. In this UNAIDS analysis, data were from 12 PMTCT studies in Burkina Faso, Côte d'Ivoire, Kenya, South Africa, Tanzania, Uganda, Zimbabwe and Botswana, reflecting >1,300 perinatally infected infants (defined by a positive PCR test before 6 weeks of age). Among untreated infants, survival was estimated by Weibull survival analysis to be 64% at 6 months, 49% at 12 months, 35% at 24 months, 25% at 36 months, 17% at 48 months, and 12% at 60 months [35–37]. To compare model-generated results to these data, we used the CEPAC-Pediatric model to simulate a cohort of infants with *in utero* or intrapartum HIV infection from birth through 60 months of age. 

#### Systematic variation in model input parameters

We anticipated that there would be substantial differences in the CD4% at birth, rate of CD4% decline, and mortality risks between children in the UNAIDS and IeDEA East Africa cohorts. To calibrate the model against UNAIDS data, we varied all CD4% and HIV-related mortality parameters, individually and in combination, applying multipliers of 0.2 to 20 to the mortality risks observed in the IeDEA cohort (Tables 2 and 3). CD4% decline was modeled to be more rapid in the first 3 months of life, based on results of the internal validation analysis. Monthly risks for clinical events (WHO3, WHO4, and TB) observed in the IeDEA cohort were similar to or greater than those reported in the literature [45–50], and were therefore not varied in calibration analyses. Non-AIDS mortality rates were also held constant, using a weighted average of UNAIDS HIV-deleted mortality data for the eight countries in the UNAIDS analysis (Table 2) [35,36,39].

**Table 3:** Systematic variations in model input parameters for calibration of CEPAC-Pediatric model.

**Parameter**	**Values**
**Initial CD4% (mean % for cohort, SD = 9%)**	
All Ages	42, 45, 47, 50
**Monthly CD4% decline at each age (%) ^a^**	
0-3 months	3.0, 4.0, 6.4 or 8.0
4-60 months	0.3, 0.5 or 1.4
**Monthly risks of clinical events (%**)	
All ages	Held equal to IeDEA clinical event risks
**HIV-deleted mortality risk (%)**	
All ages	Held equal to weighted average of HIV-deleted mortality rates from countries represented in UNAIDS cohort
**Acute mortality risk (%) ^b^**	
All Ages	0.5-5.0 X IeDEA risks (increments of 0.5)
**Chronic HIV mortality risk ^c^**	
0-6 months	1.0-20.0 X IeDEA risks (increments of 1.0)
7-12 months	1.0-20.0 X IeDEA risks (increments of 1.0)
13-24 months	0.5-5.0 X IeDEA risks (increments of 0.5)
25-36 months	0.5-5.0 X IeDEA risks (increments of 0.5)
37-48 months	0.2-2.0 X IeDEA risks (increments of 0.2)
49-60 months	0.2-2.0 X IeDEA risks (increments of 0.2)

**IeDEA**: International Epidemiologic Database to Evaluate AIDS, East African region. **m**: month.

a. Values for monthly CD4% decline reflect more rapid decline in the first three months of life than after age three months, based on published literature [43–45], and the results of internal validation analyses.

b. Acute mortality risk: risk of death within 30 days of a clinical event (WHO Stage 3, WHO Stage 4, or tuberculosis; see Methods).

c. Chronic HIV mortality: monthly risk of death for patients with no history of a clinical event, or for patients >30 days following a clinical event (see Methods). In all evaluated parameter sets, multipliers for chronic HIV mortality were limited to ranges in which multipliers applied at younger ages were ≥ multipliers at older ages. Risks were therefore permitted to remain constant or decrease (but not increase) with age. This leads to a total of 294,660 parameter combinations of chronic HIV mortality multipliers, and 141.4 million total parameter sets examined (see Methods).

Model**-**generated results were compared to empiric data in a step-wise fashion based on six key time points after birth (6, 12, 24, 36, 48, and 60 months). We first identified all combinations of CD4% values and mortality risk multipliers (Table 3) that led to model-generated mortality within 1% of the UNAIDS mortality estimate at 6 months of age (63-65%). For each of those parameter sets, multipliers were next applied to chronic HIV mortality risks for ages 7-12 months. All parameter sets producing model-generated mortality risks within 1% (absolute) of the target 12-month risk (48-50%) were retained in the next step. Chronic HIV mortality risk multipliers were then applied to ages 13-24 months; parameter sets leading to results within 1% of the 24-month target (34-36%) were retained. This process was repeated for time points of 36, 48, and 60 months. In all evaluated parameter sets, multipliers for chronic HIV mortality were limited to ranges in which multipliers applied at younger ages were greater than or equal to multipliers at older ages. Risks were therefore permitted to remain constant or decrease (but not increase) with age. Finally, all parameter sets leading to model results within these ranges were compared again to the UNAIDS mortality rates to identify all parameters sets that resulted in a RMSE <0.01%. 

## Results

### Internal model validation: Comparison of model-generated results to empiric data from IeDEA

#### Internal validation of survival

**Figure 2:**
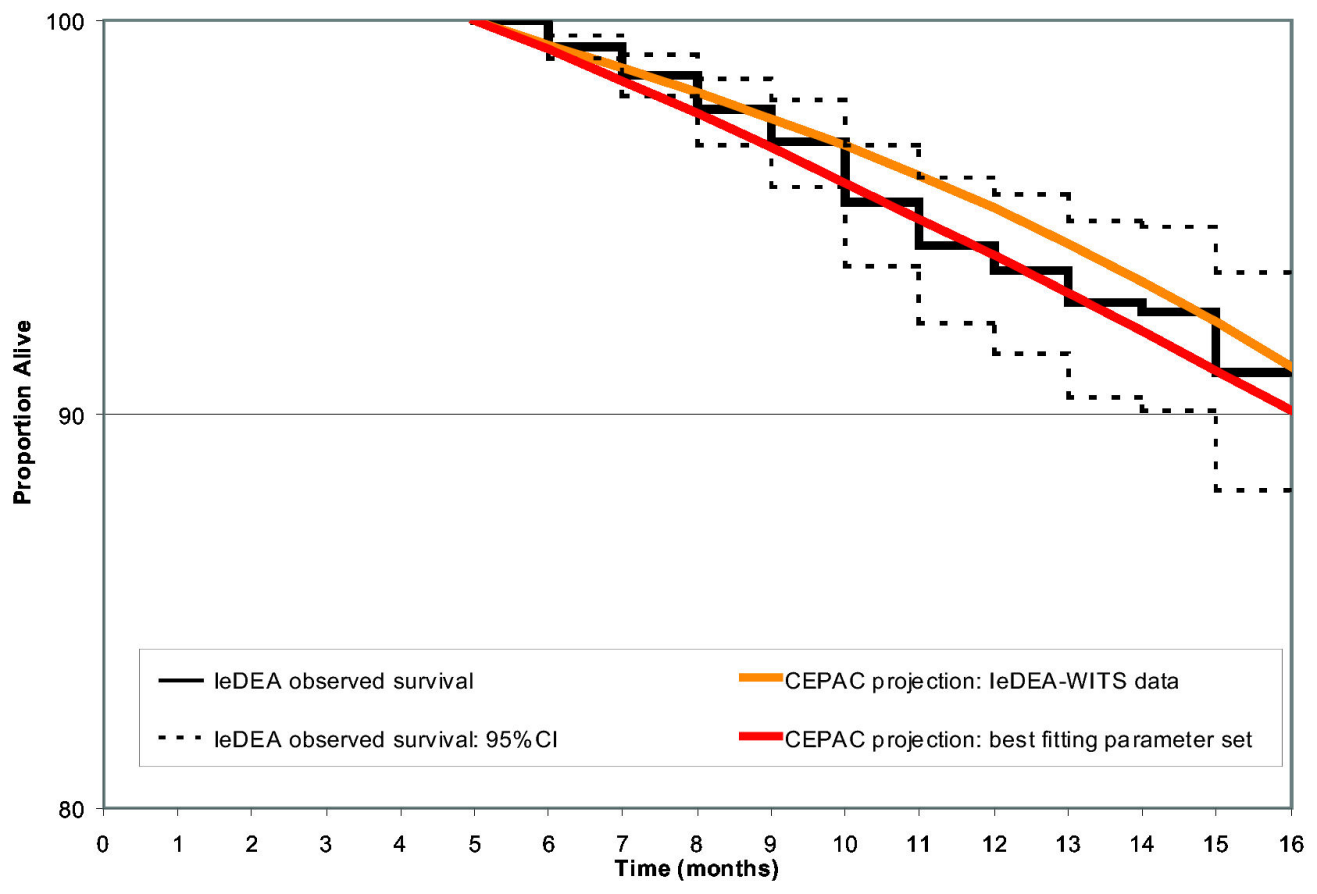
Internal validation of survival outcomes: Observed survival curves from the IeDEA East African region and projected results from the CEPAC-Pediatric Model. The solid black stepped line represents observed survival in the IeDEA cohort based on Kaplan-Meier analysis, beginning at 5 months of age. Dashed black lines reflect the upper and lower bounds of the 95% confidence intervals for IeDEA-observed survival. The orange line shows CEPAC model-projected survival using the "IeDEA-WITS projection" data (RMSE = 0.0103). The best-fitting curve is shown with the red line, reflecting mean CD4% at birth of 45.0%, CD4% decline of 6.0%/month in infants <3 months of age, and 0.3%/month for children >3 months of age (RMSE = 0.00423).

In simulations using IeDEA clinical event risk data and WITS immunologic data ("IeDEA-WITS projections"), model-projected survival (91.2% at 16 months) was slightly greater than the survival observed in the IeDEA cohort (91.1% at 16 months) (Figure 2, orange line). The RMSE for this model-generated survival curve was 0.0103, reflecting an average absolute difference of 1.03% from IeDEA observed survival. 

Systematic variation in both CD4% at birth and monthly CD4% decline led to 3,888 evaluated parameter sets, in which the RMSE between model-generated and IeDEA survival data varied widely (range, 0.00423 to 0.0798). Of these, 191 parameter sets were identified as best-fitting, with a RMSE <0.01. In general, survival was overestimated in analyses in which CD4% at birth was high and monthly CD4% decline was slow, and underestimated under the opposite conditions. The parameter set with the lowest RMSE (RMSE = 0.000423) included CD4% at birth of 45.0%, monthly CD4% decline of 6.0% in infants <3 months and monthly CD4% decline of 0.3% in children > 3 months (Figure 2, red line). 

#### Internal validation of clinical event risks

**Figure 3:**
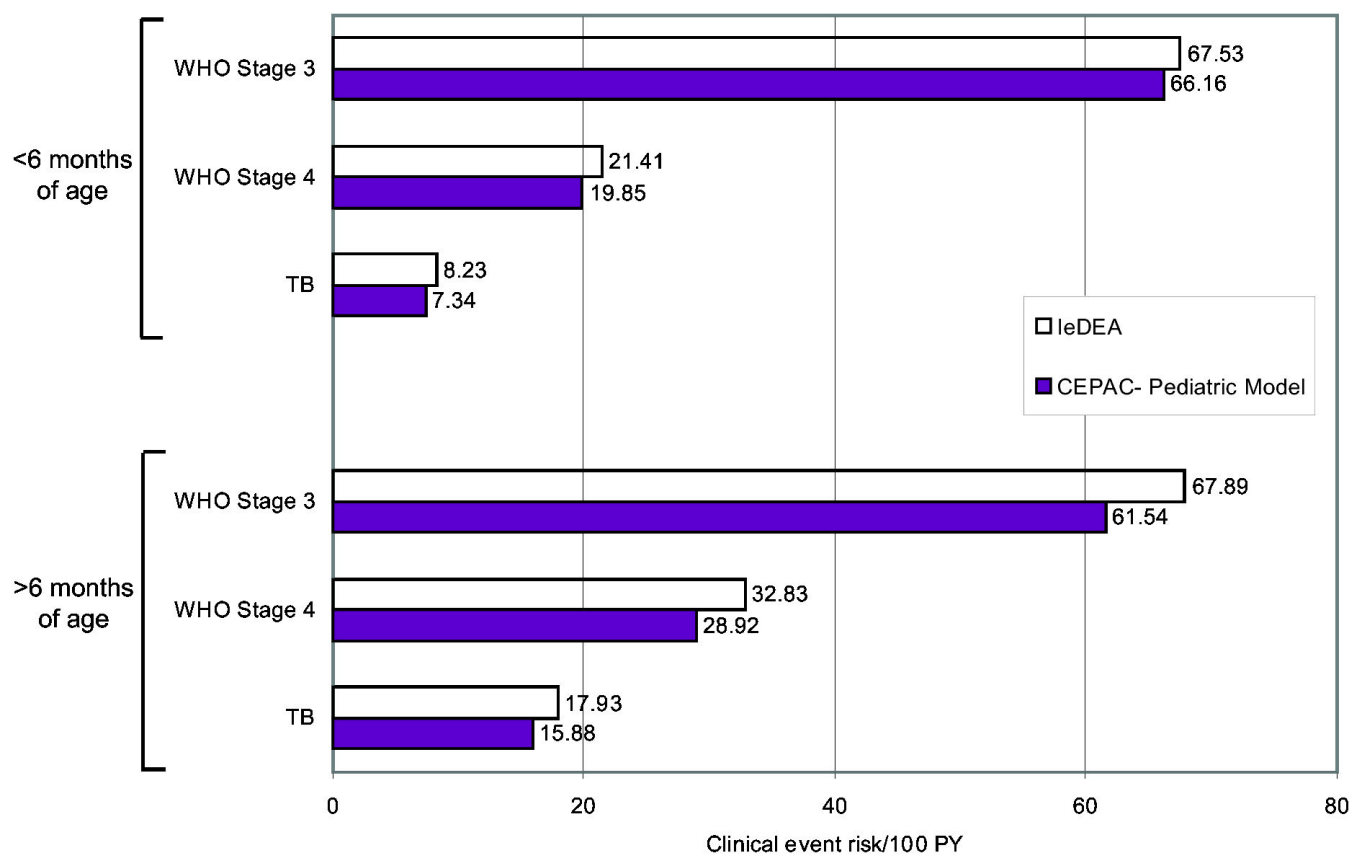
Internal validation of clinical event risk outcomes: CEPAC-Pediatric model results compared to IeDEA data. Risks of clinical events from 5-16 months of age, as observed among infants in the IeDEA East Africa region and projected by the CEPAC-Pediatric model. Simulated infants enter the model with the CD4% at birth identified in the best-fitting parameter set for the internal validation survival analyses (45.0%), and CD4% values decline as per the best-fitting parameter set (6.0%/month ages 0-3 months, 0.3%/month ages ≥3 months). Simulated infants face competing risks of all three types of clinical events, as well as "acute" and "chronic" mortality. Due to differing methods of reporting, IeDEA event risks (reported for three distinct CD4 strata) could not directly be compared to model-projected event risks (reported as a cohort average, where the cohort consists of a population with a unique distribution of CD4% each month). To generate a comparable IeDEA risk for each clinical event, we calculated an average of the three reported risks from IeDEA (CD4 <15%, CD4 15-25%, CD4 >25%) weighted by the proportion of the cohort in each CD4% strata during each month of the simulation. Model-generated rates are expected to be slightly lower than IeDEA-observed rates, due to 1) model accounting of OIs (which permits only one OI to be recorded each month), and 2) competing risks of other OIs and chronic HIV mortality in the model. **TB**: tuberculosis, **PY**: person-years.

The model also projected rates of clinical events that fit IeDEA data well. Incorporating CD4% decline rates from the best-fitting internal validation parameter sets, as well as competing clinical event and mortality risks from the IeDEA cohort, model-generated incidence rates were within 2-12% of observed IeDEA rates (Table 4, Figure 3). 

**Table 4:** Comparison of clinical event risks observed in the IeDEA East Africa cohort and. **projected by the CEPAC-Pediatric model ^*a*^** .

	**Rates/100PY**		**Monthly risk (%)**		
**Clinical event and age**	**Model generated**	**Observed in IeDEA^*b*^**	**Model generated**	**Observed in IeDEA^*b*^**	**Difference in rates (as % of IeDEA rate) ^c^**
**WHO Stage 3**					
<6m	66.16	67.53	5.36	5.47	2.0
≥6m	61.54	67.89	5.00	5.50	9.4
**WHO Stage 4**					
<6m	19.85	21.41	1.64	1.77	7.3
≥6m	28.92	32.83	2.41	2.70	11.9
**Tuberculosis**					
<6m	7.34	8.23	0.61	0.68	10.8
≥6m	15.88	17.93	1.32	1.48	11.5

**PY**: person-years; **m**: months

a. As described in the Methods, patients enter the model with CD4% at birth from the best-fitting parameter set in the internal validation survival analyses (45.0%). CD4% values decline as per the best-fitting parameter set (6.0%/month ages 0-3 months, 0.3%/month ages >3 months). Simulated infants face competing risks of all three types of clinical events, as well as "acute mortality" and "chronic HIV mortality.”

b. Due to differing methods of reporting, IeDEA event risks (reported for three distinct CD4 strata) could not directly be compared to model-projected event risks (reported as a cohort average, where the cohort consists of a population with a unique distribution of CD4% each month). To generate a comparable IeDEA risk for each clinical event, we calculated an average of the three reported risks from IeDEA (CD4 <15%, CD4 15-25%, CD4 >25%) weighted by the proportion of the cohort in each CD4% strata during each month of the simulation.

c. Model-generated rates are expected to be slightly lower than IeDEA-observed rates, due to: 1) Model accounting of clinical events (which permits only one event to be recorded each month), and 2) competing risks of other events and “chronic HIV mortality” in the model.

### Model calibration: Comparison of model-generated results to published UNAIDS pre-ART survival curves

**Figure 4:**
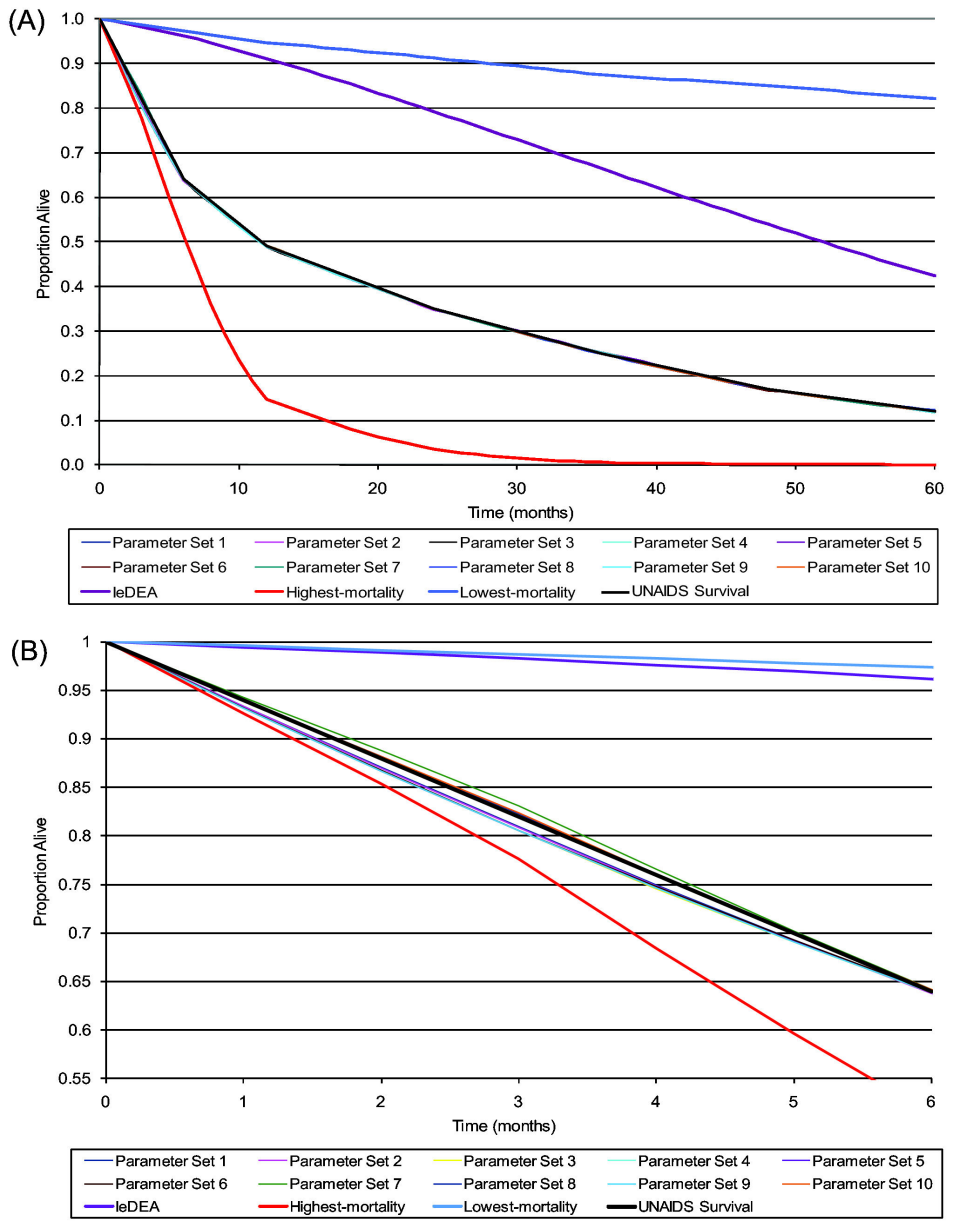
CEPAC-Pediatric model calibration analyses: Projected survival (A). Model-projected survival curves from age 0-60 months for: 1) Base-case IeDEA mortality data used in the internal validation analyses (purple line); 2) the empiric UNAIDS mortality data (black line); 3) 10 of the best-fitting parameter sets (with the lowest RMSE) identified in the calibration analyses (group of colored lines surrounding and almost completely overlapping with the black UNAIDS line); 4) the lowest-mortality risk parameter set from Table 3 (blue line) and 5) the highest-mortality risk parameter set from Table 3 (red line). The 10 sample best-fitting parameter sets from calibration analyses are almost entirely obscured by the UNAIDS survival data (black line) due to their extremely close fit to the calibration target. The IeDEA survival curve from internal validation analyses, and both the highest- and lowest-mortality risk parameter sets are all projected to 60 months of age for comparison only, as they did not meet the threshold of UNAIDS risk ±1% at 6 months and therefore were not formally evaluated at subsequent time points in the calibration analyses. **B**: A zoom plot, enlarging the results for months 0-6, shows the nearly-overlapping curves in larger detail.

We examined 141 million parameter sets (all possible combinations from Table 3). We identified 9,943 best-fitting parameter sets through the step-wise selection process, in which we retained only parameter sets that led to model-generated mortality risks within ±1% of UNAIDS survival risks at key time points (RMSE <0.01). Projected survival for the 10 best-fitting parameter sets with the lowest RMSE is shown in Figure 4: UNAIDS survival and is depicted as the black line and all model-generated survival curves overlap almost entirely with the UNAIDS survival curve. Figure 4 also shows projections using the IeDEA survival from the internal validation analyses, to illustrate the lower mortality seen in the IeDEA cohort (green line), as well as the highest- and lowest-mortality risk parameter sets from Table 3 (red and blue lines) to show the range that the model is capable of generating. 

The individual components of 10 of the best-fitting parameter sets with the lowest RMSE are shown in Table 5. The data parameters requiring the largest increase in risk to match UNAIDS survival data were acute mortality within 30 days of a clinical event (multipliers applied to IeDEA event risks ranged from 4-5 in all parameter sets), and chronic HIV mortality in the first 12 months of life (multipliers of 14-17 for infants ages 0-6 months, and 4-8 for ages 7-12 months). After 48 months of age, calibrated chronic HIV mortality risks were slightly lower than IeDEA data (multipliers of 0.4-0.8).

**Table 5:** Root-mean-squared error for key parameters sets in the calibration of the CEPAC-Pediatric model to UNAIDS survival data.

			**Chronic HIV mortality multiplier ^*c*^**						
**Mean CD4% at birth**	**Monthly CD4% decline ^*a*^**	**Acute clinical event mortality multiplier ^*b*^**	**0-6m**	**7-12m**	**13-24m**	**25-36m**	**37-48m**	**49-60m**	**Root-mean-squared error (RMSE) ^*d*^**
**10 best-fitting parameter sets**									
45	4, 0.5	4	17	7	3	1.5	1.2	0.4	0.00122
42	3, 0.5	4	17	7	3	1.5	1.2	0.4	0.00146
50	4, 0.5	5	17	8	3.5	2	1.6	0.4	0.00152
47	3, 0.5	5	17	8	3.5	2	1.6	0.4	0.00162
50	4, 0.5	5	17	8	3.5	2	1.6	0.6	0.00172
50	4, 0.5	5	17	8	3.5	2	1.8	0.4	0.00173
47	8, 0.3	4	14	4	1.5	1	1	0.6	0.00174
45	6.4, 0.3	4	15	5	2	1.5	1.4	0.6	0.00176
47	3, 0.5	5	17	8	3.5	2	1.6	0.6	0.00176
45	6.4, 0.3	4	15	5	2	1.5	1.4	0.8	0.00181
**Base-case IeDEA survival from internal validation analysis, projected to 60 months of age**									
45	6, 0.3	1	1	1	1	1	1	1	0.383
**Lowest-mortality risk parameter set from Table 3, projected to 60 months of age**									
50	3, 0.3	0.5	1	1	0.5	0.5	0.2	0.2	0.575
**Highest-mortality risk parameter set from Table 3, projected to 60 months of age**									
42	8, 1.4	5	20	20	5	5	2	2	0.236

a. CD4% decline is shown as monthly decline (in CD4 percentage points) for months 1-3 of life, followed by for months 4+ of life.

b. Multipliers were applied to the monthly risks of "acute mortality" derived from the IeDEA cohort (defined as mortality <30 days following a WHO3, WHO4, or TB clinical event).

c. Multipliers were applied to monthly risks of "chronic HIV mortality" derived from the IeDEA cohort (defined separately as mortality risks among infants with no history of clinical event, or >30 days after a clinical event for infants with a history of clinical event).

d. Root-mean-squared error of CEPAC-Pediatric model projections compared to UNAIDS survival data at 6, 12, 24, 36, 48, and 60 months of age. RMSE is calculated by 1) calculating the difference between observed and projected survival proportions at each time point, 2) squaring these six absolute differences, 3) averaging the squared values, and 4) taking the square root of this average value. RMSE reflects an average difference between observed and projected survival (as a percent) over the six time points.

## Discussion

We developed a patient-level computer simulation model of disease progression among perinatally HIV-infected infants -- the CEPAC-Pediatric model. This represents the first description of a Monte Carlo microsimulation model of untreated HIV disease in children, and the most detailed description to date of internal validation and calibration of a model of pediatric HIV disease according to recommended practices [15–17]. 

In internal validation analyses, using a single set of input parameters to assess model structure, model outputs closely matched empiric data from the IeDEA East African regional pediatric cohort. Model-projected survival was closest to empiric survival data if the immunologic data parameters (not available from IeDEA) included an average CD4% at birth of 45%, a CD4% decline of 6%/month for infants < 3 months of age, and a CD4% decline of 0.3%/month for children >3 months of age. When model-projected clinical event risks were compared to empiric IeDEA event risks, model results matched observed data reasonably well. The difference between model-generated results and observed data ranged from 2-12%, less than the 10-15% criterion accepted as "good-fitting" in a prior study [5].

In calibration analyses, we identified new values for model input parameters that allowed our projections to match more generalizable survival data from untreated, African children. Large increases in IeDEA-observed mortality risks were required in the first 12 months of life for model projections to match UNAIDS survival data [35–38]. There are likely to be at least two reasons for this finding. First, data in the UNAIDS analysis were collected before widespread pediatric ART availability (in only one included study did any HIV-infected children initiate ART) [36]. In contrast, children in IeDEA were likely to initiate ART when needed; although they were then censored from our analysis, this likely averted many of the risks of opportunistic infection and death that would have occurred had no ART been available. 

Second, the IeDEA dataset may reflect some degree of “survivor bias:” children who survive the first months and years of life may be longer-term survivors, with slower disease progression than those who become ill in infancy and are unavailable to enroll in many cohort studies. Infants in the UNAIDS analysis were followed from birth, permitting early infant mortality to be observed and avoiding this survivor bias, whereas infants in the IeDEA cohort enrolled at a median of 5 months of age. Our finding that IeDEA-observed mortality risks in the first 12 months of life required the largest increases to match UNAIDS data suggests that unobserved pre-enrollment mortality was likely a key explanation for the overall very low mortality observed in IeDEA. Such survivor bias has also been described in other cohorts of HIV-infected children, in which median age at enrollment is >1 year, and often up to 5 years [47,51–55]. In studies that have enrolled children at younger ages, mortality is reported to be two- to eight-fold greater among infants <12 months of age compared to older children [46,47,53,56]. Consistent with this literature, several good-fitting parameter sets in our model calibration analyses required chronic HIV mortality risks after 48 months of age to be reduced below the risks observed in the IeDEA cohort to match UNAIDS data. This suggests that mortality risks for children who have survived to age 4 may be less than the risks among younger children in the IeDEA cohort. Empiric data on CD4% stratified risks of acute clinical events and mortality for untreated, HIV-infected children ages 2-5 years are limited; in their absence, model-calibrated mortality estimates can inform the impact of deferred ART initiation in children of these ages.

This analysis has several limitations. First, data to completely parameterize the model were not available from IeDEA, and immunologic data from the US-based WITS cohort were used where IeDEA data were unavailable [33]. Due to the lack of an independent source of data, we were unable to perform a true validation of the model, and instead first performed an internal validation of IeDEA OI and mortality risks and then separately calibrated the model to fit the UNAIDS survival curves. However, the flexibility to incorporate data from a variety of sources and to evaluate the impact of these heterogeneous data sources is also a strength of modeling analyses [13]. The WITS-derived CD4% inputs were varied extensively in sensitivity analyses, and found to have modest impact on goodness-of-fit between model-generated and observed survival risks. 

Second, there is no single accepted criterion by which to compare model-generated results to empiric data. Instead, expert guidance recommends that investigators choose and explicitly describe a criterion that fits their model structure and data sources [5,23–25,30,57]. We chose RMSE to assess goodness-of-fit between projected and observed survival. The complex microsimulation structure of the CEPAC model renders Bayesian analysis computationally infeasible [58], empiric datasets were too small to permit separate analyses in training and validation sets. We therefore selected a method that is transparent, well-described in policy models, and comparable to methods used to validate the adult CEPAC model [19]. Although some possible parameter sets were necessarily excluded with this approach, we sampled the parameter space systematically, varying all model parameters in small increments through clinically plausible ranges and considering all 141 million resulting parameter combinations. 

In summary, we report the development, internal validation, and calibration of the CEPAC-Pediatric natural history model of HIV disease progression in young children. The model demonstrates excellent performance in internal validation analyses to evaluate model structure. The model also permits wide-ranging sensitivity analyses on all key input parameters, and variations in key clinical and immunologic parameters lead to model-generated survival curves that calibrate closely to UNAIDS survival data for untreated, perinatally HIV-infected children. Differences in the mortality risk parameters required to match data from the IeDEA cohort (model internal validation) and the UNAIDS cohort (model calibration) highlight the importance of early infant mortality before enrollment in many cohort studies, as well as the ability of simulation models to estimate mortality risks among children ages two to five in the absence of such empiric data. This validated CEPAC-Pediatric model will be well-suited to address critical policy questions in pediatric HIV care for children from birth through five years of age.
